# Glucose and Fructose Supplementation and Their Acute Effects on Electrocardiographic Time Intervals during Anaerobic Cycling Exercise in Healthy Individuals: A Secondary Outcome Analysis of a Double-Blind Randomized Crossover-Controlled Trial

**DOI:** 10.3390/nu14163257

**Published:** 2022-08-09

**Authors:** Max L. Eckstein, Paul Zimmermann, Maximilian P. Erlmann, Nadine B. Wachsmuth, Sandra Haupt, Rebecca T. Zimmer, Janis Schierbauer, Daniel Herz, Felix Aberer, Harald Sourij, Barbara Obermayer-Pietsch, Othmar Moser

**Affiliations:** 1 Division of Exercise Physiology and Metabolism, BaySpo—Bayreuth Center of Sport Science, University of Bayreuth, 95440 Bayreuth, Germany; 2 Department of Cardiology, Klinikum Bamberg, 96049 Bamberg, Germany; 3 Cardiovascular Diabetology Research Group, Division of Endocrinology and Diabetology, Department of Internal Medicine, Medical University of Graz, 8036 Graz, Austria; 4 Endocrinology Lab Platform, Division of Endocrinology and Diabetology, Department of Internal Medicine, Medical University of Graz, 8036 Graz, Austria

**Keywords:** glucose metabolism, heart rate variability, premature ventricular complexes, heart rate turn point, electrocardiography, fructose, sucralose

## Abstract

The impact of glucose and fructose supplementation on acute cardiac effects during cardiopulmonary exercise testing (CPET) is a topic that is rarely investigated. The aim of the presented secondary outcome analysis of a double-blind, randomized crossover-controlled trial was to investigate the impact of glucose (Glu), fructose (Fru), glucose and fructose (GluFru), and sucralose on electrocardiogram (ECG), heart rate variability (HRV), premature ventricular complexes (PVCs), and heart rate turn points (HRTP) during CPET. Fourteen healthy individuals (age 25.4 ± 2.5 years, body mass index (BMI) 23.7 ± 1.7 kg/m^2^, body mass (BM) of 76.3 ± 12.3 kg) participated in this study, of which 12 were included for analysis. Participants received 1 g/kg BM of Glu, 1 g/kg BM of Fru, 0.5 g/kg BM of GluFru (each), and 0.2 g sucralose dissolved in 300 mL 30 min prior to each exercise session. No relevant clinical pathology or significant inter-individual differences between our participants could be revealed for baseline ECG parameters, such as heart rate (HR) (mean HR 70 ± 16 bpm), PQ interval (146 ± 20 ms), QRS interval (87 ± 16 ms) and the QT (405 ± 39 ms), and QTc interval (431 ± 15 ms). We found preserved cardiac autonomic function by analyzing the acute effects of different Glu, Fru, GluFru, or sucralose supplementation on cardiac autonomic function by Schellong-1 testing. SDNN and RMSSD revealed normal sympathetic and parasympathetic activities displaying a balanced system of cardiac autonomic regulation across our participating subjects with no impact on the metabolism. During CPET performance analyses, HRV values did not indicate significant changes between the ingested drinks within the different time points. Comparing the HRTP of the CPET with endurance testing by variable metabolic conditions, no significant differences were found between the HRTP of the CPET data (170 ± 12 bpm), Glu (171 ± 10 bpm), Fru (171 ± 9 bpm), GluFru (172 ± 9 bpm), and sucralose (170 ± 8 bpm) (*p* = 0.83). Additionally, the obtained time to reach HRTP did not significantly differ between Glu (202 ± 75 s), Fru (190 ± 88 s), GluFru (210 ± 89 s), and sucralose (190 ± 34 s) (*p* = 0.59). The significance of this study lies in evaluating the varying metabolic conditions on cardiac autonomic modulation in young healthy individuals. In contrast, our participants showed comparable cardiac autonomic responses determined by ECG and CPET.

## 1. Introduction

Globally, cardiovascular diseases represent the leading cause of death, and in developed and developing countries their prevalence has been emerging incessantly over the last decades [1,2]. Though cardiovascular disease prognosis and associated complications have improved over the years due to better medical care, scientific research on prognostic factors remains essential [2,3].

Heart rate response during exercise, heart rate variability (HRV), premature ventricular complexes (PVC), QT interval, standard deviation of R-R intervals (SDNN), and root mean square of successive differences between normal heartbeats (RMSSD) represent relevant electrocardiographic parameters and minor alterations can potentially contribute to life-threatening cardiac arrhythmias, sudden cardiac death (SCD), and other cardiovascular events [2,4]. In this context, sociodemographic and clinical characteristics, such as body composition and anthropometric data, dietary patterns, and physical activity have been reported for their different impact on HRV [2,5,6,7]. Adverse associations between cardiac function impairment, i.e., reduced HRV and cardiac ectopic beats, and different doses of alcohol, obesity, and the waist-to-hip ratio have been demonstrated in previous research [5,6,7,8,9]. Previous studies have also shown an imbalance in autonomic cardiac modulation following a sudden increase in plasma glucose concentration and hyperinsulinaemia—whereby subjects with metabolic disease were mainly affected [4]. When investigating the influence of energy drinks on heart rhythm, especially supraventricular extrasystoles (SVES), mean heart rate (HR), and electrocardiographic time intervals—0 preferable QT intervals—they were found to increase the rate of SVES, but they had no effect on QT intervals [10].

In recent decades, HRV—serving as a measure for cardiac autonomic function—has been intensively studied for risk stratification in healthy subjects as well as in subjects with concomitant cardiac disease such as heart failure, heart rhythm diseases, or previous myocardial infarction [2,11]. For these cardiac-affected individuals, reduced HRV has been identified as a predictor of increased risk of cardiovascular mortality and cardiovascular events [12]. To date, it is not entirely clear whether reduced HRV impacts the onset of harmful adverse cardiac events or whether reduced HRV indicates the presence of these diseases [2,12].

Although studies have suggested a specific association between dietary and lifestyle patterns with HRV and cardiac ectopic beats, the scientific evidence is limited and discussed controversially [2]. Given the prognostic relevance of HRV on cardiac ectopic beats and premature ventricular complexes (PVC), it is crucial to understand the interaction between heterogeneous environmental and alimentary factors and the clinical arrhythmogenic impact [2]. The presented exploratory randomized crossover-controlled trial aimed to elucidate the association between glucose and fructose supplementation on acute cardiac response in healthy individuals. In particular, HRV, PVC, QT interval analysis, SDNN, RMSSD, and HRTP curves were analyzed during cardiopulmonary exercise testing (CPET) to investigate the prognostic relevance of different cardiac responses in individuals undergoing different supplementation. The findings of this study might prove the feasibility of different combined carbohydrate supplementations in healthy individuals and assess their specific cardiometabolic relevance and potential to induce proarrhythmogenic alterations in healthy individuals.

## 2. Materials and Methods

This was a single-center, randomized, double-blind crossover-controlled secondary outcome analysis, assessing the effects of Glu, Fru, GluFru, and sucralose on cardiac performance during CPET in healthy, physically active individuals. The local ethics committee of the University of Bayreuth (Bayreuth, Germany) approved the study protocol (O 1305/1. GB 15 November 2021), and the trial was registered at the German Clinical Trials Register (DRKS00027153). The study was conducted in conformity with the declaration of Helsinki and Good Clinical Practice. Before any trial-related activities, potential participants were informed about the study protocol, and participants gave their written informed consent to participate in this trial. This study is designed as a proof-of-concept study.

### 2.1. Eligibility Criteria

Eligibility criteria included male or female individuals aged 18–65 years with a body mass index (BMI) of 18.0–29.9 kg/m^2^, both inclusive. Participants with a normal glucose tolerance, measured via overnight fasting blood glucose (BG) levels, were included. Individuals were excluded if they were enrolled in a different study, received investigational medicinal products, had a supine blood pressure outside of the range of 90–150 mmHg for systolic and 50–95 mmHg for diastolic after resting for five minutes in a prone position. Furthermore, participants were excluded if they had a history of multiple and/or severe allergies or intolerances to any trial-related products. A further exclusion criterion was the regular or irregular intake of any medication with a potential impact on the parameters assessed (blood pressure lowering therapy, antiarrhythmic drugs, antidepressants with QT prolonging potential). A medical investigator assessed inclusion and exclusion criteria at the screening visit before enrolment in the study.

### 2.2. Study Design

After inclusion in the study, participants were assigned to ascending numbers. They were then allocated to the order in which the trial visits were conducted in a cross-over randomized fashion with the software Research Randomizer 4.0 (Social Psychology Network, Lancaster, PA, USA)^®^ (1:1:1:1) [13]. For the study visits, participants received either 1 g/kg body mass (BM) Glu (Fisher Scientific, Loughborough, UK), 1 g/kg BM Fru (Grüssing, Felsum, Germany), and 0.5 g/kg BM of GluFru (each) dissolved in 300 mL water. Sucralose (MyProtein, Norwich, UK) was administered as a fixed amount of 0.2 g per dosage to mimic the taste of the other trial products. The artificial sweetener was used as a placebo control to imitate the taste of the other study-related products to avoid selection bias. Between each visit, a minimum period of 48 h was maintained.

#### 2.2.1. Screening Visit

Participants were instructed about all study-related procedures during the screening visit. Subsequently, medical staff asked them about their medical history. Anthropometric parameters were assessed via bioelectrical impedance analysis (Inbody 720, Inbody Co., Seoul, Korea) for body composition and via manual measurement for body height (Seca 217, Seca, Hamburg, Germany). In addition, a Schellong-1 test was conducted in which participants were asked to rest in a supine position for 10 min, stand up as rapidly as possible and remain standing for two additional minutes. During this test, participants received a Holter-electrocardiograph (Holter ECG) (Faros 180; Bittium, Oulu, Finland) to monitor HRV and cardiovagal response to the change in body position. Furthermore, blood pressure was measured at the beginning, after standing up, and after two minutes of standing. This orthostatic test is a simple clinical function test that ensures an adequate heart rate and blood pressure response and detects potential orthostatic disturbances. A 12-lead ECG (CardioPart 12, Amedtec, Aue-Bad Schlema, Germany) was recorded subsequently, and a cardiac assessment (blood pressure, heart rate) was performed. A capillary blood sample was taken from a hyperemized earlobe to ensure blood glucose levels were within the physiologic range for healthy adults. During CPET, heart rate was measured continuously via a 12-lead ECG for safety reasons. Throughout CPET, breath-by-breath measurements were conducted and averaged over 5 s for later analysis (METALYZER^®^ 3B; Cortex Biophysik GmbH, Leipzig, Germany).

At the beginning of the incremental CPET, participants sat on the cycle ergometer for 3 min (0 W) before starting the warm-up period of 3 min by pedaling at a workload of 20 W. Then, the workload increased by 15 W (female) or 20 W (male) every minute, depending on their expected functional capacity to reach a total test duration of 8 to 12 min. Finally, a 3 min active recovery was conducted at 20 W followed by a 3 min passive recovery (0 W). The first and second ventilatory threshold (VT_1_ and VT_2_), as well as the maximum power output (P_max_), were determined to prescribe the exercise intensity for the upcoming four exercise sessions.

#### 2.2.2. Trial Visits

Participants were asked to visit the laboratory in the morning after an overnight fast (>12 h) which allowed only water to be ingested. Prior to the start of the visit, BM was measured to ensure no significant increases or decreases occurring between visits. Participants then received an opaque shaker bottle filled with a solution of 300 mL water and their individual amount of either Glu, Fru, GluFru, or sucralose. All beverages were prepared by a researcher not otherwise involved in the trial. Shakers were labelled with the participants’ study ID to avoid distribution errors. Drinks were given to the participants directly from the study team. Prior to the consumption of the drinks, participants received a Holter-ECG to measure HRV. Participants were asked to consume the beverage as quickly as possible and remain in an upright sitting position for the following 24 min. During that time, participants were equipped with a 12-lead ECG for the detection of heart rate curves and spirometric device for breath-by-breath analysis. The exercise test on the cycle ergometer was initiated with a 3 min resting period followed by a 3 min warm-up at 20 W. In the next period, the power output increased to 120% of VT2, and participants were verbally motivated to cycle until volitional exhaustion. Volitional exhaustion was defined as the individual inability to keep the cadence below 60 rpm. Afterwards, participants were asked to remain seated in an upright position on the cycle ergometer for 3 min. Between visits, a minimum break of 48 h during which participants were asked to refrain from excessive physical activity was adhered to. The timing for the consumption of the beverages and the start of exercise was based upon a preliminary study conducted by our research group to detect peak hormonal and glycemic responses prior to the start of exercise [14,15].

### 2.3. HRV Measurement

The Holter monitor used in this study applied one channel with a 1000-Hz sampling rate. The HRV measures evaluated in the time domain analysis included standard deviation of R-R intervals (SDNN), square root of the mean standard difference of successive R-R intervals (RMSSD), and percentage of pairs of R-R intervals with >50 ms difference (pNN50%). Power spectral analysis for the analysis of the frequency domain was conducted via Fast-Fourier Transformation in Cardiscope (Hasiba Medical GmbH, Graz, Austria). Low frequency/high frequency (LF/HF) and RMSSD are measures of the balance between parasympathetic and sympathetic activity. HRV values were assessed according to the guidelines published by the Task Force of the European Society of Cardiology (ESC) and the North American Society of Pacing and Electrophysiology (NASPE) for the assessment of HRV [16,17].

### 2.4. Heart Rate Curves

The non-invasive anaerobic threshold was defined by the HRTP. HRTP was demarcated as the intersection of two regression lines of heart rate performance curves (HRPC) between post-warm-up and P_max_, determined from a second-degree polynomial representation satisfying the condition of least error squares [18]. For analysis during the endurance exercise tests, HRTP was measured the same way that it was measured during the exercise tests to investigate the heart rate response to a constant overload 20% above the VT_2_.

### 2.5. ECG Analysis

The ECGs of participating subjects were evaluated for resting heart parameters, such as baseline heart rate at rest, measured in beats per minute (bpm); PQ interval analysis, measured in ms; QRS interval assessment, measured in ms; and QT and QTc interval recording, displayed in ms. The HR, QRS and T axis, PQ and QT intervals, and QRS duration were analyzed digitally by the ECG software of Amedtec (CardioPart 12, Amedtec, Aue-Bad Schlema, Germany). The obtained ECG results were additionally analyzed for criteria of left ventricular (LV) hypertrophy or increased LV mass, which represent physiological cardiac remodeling due to chamber enlargement, increased wall thickness, or increased volume, by assessment of the following indexes: the Sokolow Lyon index, estimated by R wave (V1/V2) + S wave (V5/V6) ≥ 35 mm and the Cornell Index, estimated by R wave (aVL) + S wave (V3) ≥ 28 mm [19]. All subjects were analyzed for conspicuous ECG features, such as sinus bradycardia (defined as HR < 60 bpm), first and second (Mobitz I) degree atrioventricular blocking, QRS complex widening (defined as QRS complex > 120 ms), left and right axis deviation (more negative than 0° or more positive than 110°), early repolarization (ER) pattern, and criteria for preexcitation syndromes [20]. No subject had to be excluded because of 12-lead ECG abnormalities.

### 2.6. Statistics

All data were assessed for normal distribution by means of the Shapiro–Wilk normality test. Statistical differences were analyzed either via mixed-effects model or with the Geisser–Greenhouse correction. Tukey’s multiple comparisons tests with individual variances were computed for each comparison if necessary. Results are presented as mean ± standard deviation. Statistical significance was accepted at *p* ≤ 0.05.

## 3. Results

A total of 14 adults (9 females) were included in the study (age 25.4 ± 2.5 years, BMI 23.7 ± 1.7 kg/m^2^, with a BM of 76.3 ± 12.3 kg). Two participants had to be withdrawn from the analysis. One individual had an episode of asymptomatic ventricular extrasystoles with a 4:1 ratio during one cycling test which might trigger ventricular arrhythmias during the test as judged by the medical investigator. The other individual did not perform the CPET at the screening visit until volitional exhaustion which is why the following exercise tests were conducted at an incorrect LTP_2_, leading to an outlier that had to be excluded from the analysis. Hence, 12 data sets were included for analysis. Anthropometric data and results of the 12-lead resting ECG are displayed in Table 1. 

### 3.1. Anthropometry

Participants included (*n* = 12, 9 females, 23.2 ± 1.0 years, BMI, 22.3 ± 1.3 kg/m^2^ with a BM of 67.3 ± 7.7 kg) had an empty cardiac history and did not take any relevant medication.

All participants enrolled in the study showed similar results following 12-lead ECG measurements at baseline (Table 1). Therefore, the resting HR did not reveal any significant bradycardia (mean HR 70 ± 16 bpm). The PQ measurements (146 ± 20 ms), QRS interval measurements (87 ± 16 ms), and the QT and QTc interval assessments (405 ± 39 ms; 431 ± 15 ms) did not reveal any relevant clinical pathology and significant inter-individual differences between our participating subjects. The analyzed and obtained data represented findings in healthy subjects without any pathological findings, essentially preexcitation syndromes or QT interval alterations caused by medication or electrolyte disturbances. All participating subjects displayed no severe abnormalities in the 12-lead ECG resting parameters, atrioventricular blockings, any signs of ER pattern, or preexcitation syndrome pattern. The 12-lead ECG results were correlated for LV hypertrophy based on the voltage criteria indexes, the Sokolow Lyon Index and the Cornell index, whereby no severe ECG criteria could be revealed for relevant LV hypertrophy in any subject based on the ECG analyses. Analyzing the QRS axis, no significant QRS widening or blocking was detected, whereas the QRS axis showed no relevant left or right axis deviation, and the indifferent or vertical axis location type was equally prevalent among our participating subjects.

**Figure 1:**
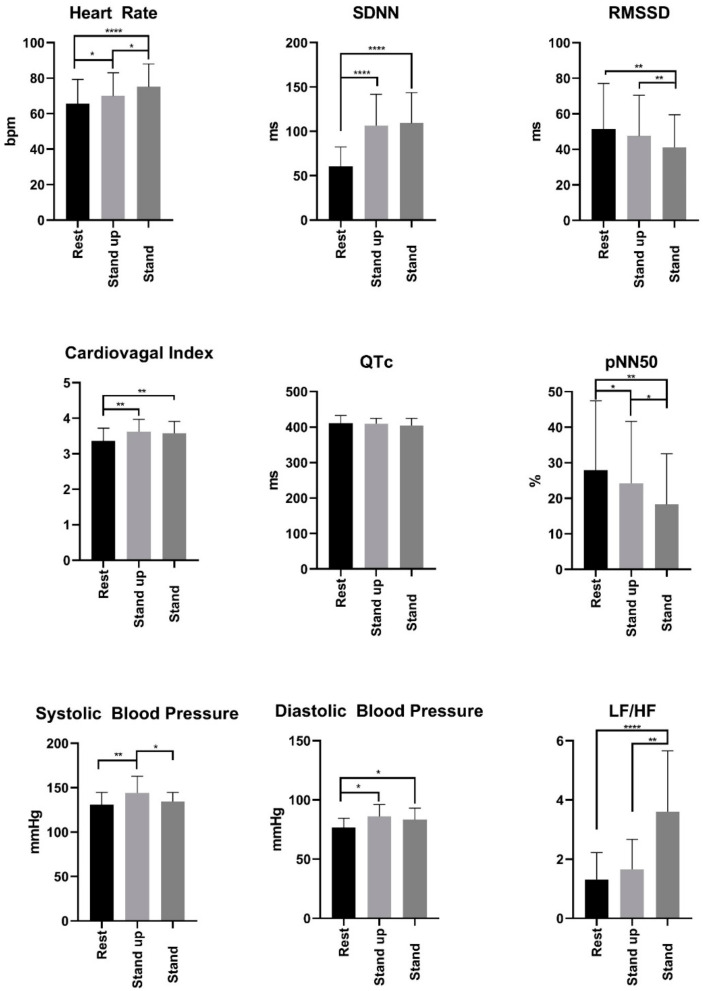
HRV detected during Schellong-1 at rest. Stars indicate level of significance. * indicates *p* < 0.05; ** indicates *p* < 0.01; **** indicates *p* < 0.0001; SDNN: standard deviation of adjacent NN intervals; RMSSD: root mean square of adjacent NN intervals; QTc: corrected QT time; pNN50: percentage of NN pairs that deviate by more than 50 ms; LF/HF: ratio between low frequency and high frequency.

The results of analyzing the acute effects of different Glu, Fru, GluFru, or sucralose supplementation on cardiac autonomic function by Schellong-1 testing are presented in Figure 1. These results were analyzed by Schellong-1 testing to assess cardiac autonomic function due to its impact on HR, diastolic and systolic blood pressure measurement, and HRV measurements. The subjects’ HRV data were collected over three periods: 10 min in a sitting position after supplementation of the specific beverage, during the fast stand up, and the following standing period. The subjects’ HR, diastolic, and systolic blood pressure reactions displayed a preserved cardiac autonomic function independent of the administered solution through a small HR and blood pressure increase during the standing period as compensatory sympathetic regulation. The displayed QT intervals revealed no significant differences across our participants and different types of supplemented beverages. Comparing the SDNN values in the standing to the resting positions, significantly higher values were found, whereas the RMSSD parameters showed an inverse trend. Both parameters reveal normal sympathetic and parasympathetic nervous activities displaying a balanced system of cardiac autonomic regulation across participating subjects with no impact from the individual supplementation.

**Figure 2:**
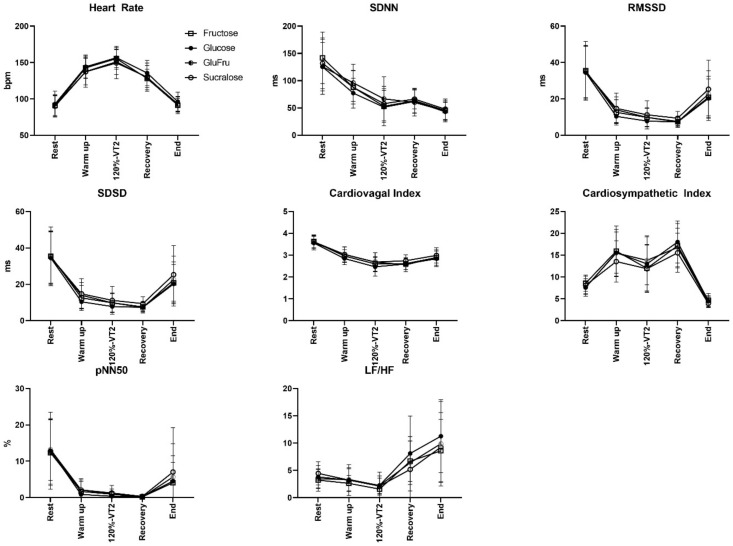
HRV variables during exercise test at rest, minute 1 of the warm-up, minute 1 of the exercise at 120% of the VT_2_ (4 min), during recovery directly after the end of exercise, and 30 min after the end of the exercise. Open squares indicate fructose (Fru), full circles indicate glucose (Glu), half-circles indicate GluFru, and open circles indicate sucralose. SDNN: standard deviation of adjacent NN intervals; RMSSD: root mean square of adjacent NN intervals; QTc: corrected QT time; SDSD: related standard deviation of successive RR interval differences; pNN50: percentage of NN pairs that deviate by more than 50 ms; LF/HF: ratio between low frequency and high frequency.

The plasma glucose levels in response to individual supplementation amounts and their accompanying cardiac autonomic modulation were evaluated for HR response, SDNN, RMSSD, LF/HF, and the displayed parameters in Figure 2.

Analyses of the HRV data did not indicate significant changes of HRV values between the different beverages during any time point of analyses. After the Glu, Fru, GluFru, or sucralose loading, our participants showed comparable results across the estimated parameters, suggesting balanced autonomic cardiac response with increased sympathetic and decreased parasympathetic tone. These results in ECG assessment display comparable preserved cardiac autonomic modulation due to variable supplemented metabolic conditions.

### 3.2. Heart Rate Turn Point (HRTP) Analysis

**Figure 3:**
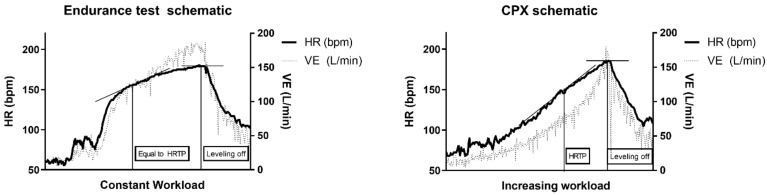
Schematic of the endurance exercise test at 120% VT_2_ measured via 12-lead ECG and spirometry in comparison to the CPX test. VE: ventilation; HRTP: heart rate turn point; HR: heart rate.

When the HRTP of the CPET performance data was compared to the endurance CPET performance data with supplementation, no significant differences were found between the HRTP of the CPET data (170 ± 12 bpm), Glu (171 ± 10 bpm), Fru (171 ± 9 bpm), GluFru (172 ± 9 bpm), and sucralose (170 ± 8 bpm) (*p* = 0.83). The turn point was reached following the ingestion of Glu after (202 ± 75 s), Fru (190 ± 88 s), GluFru (210 ± 89 s), and after sucralose (190 ± 34 s) (*p* = 0.59). Schematics for the test procedures and run of each exercise test are shown as a schematic in Figure 3.

## 4. Discussion

This secondary outcome analyses of a double-blind randomized crossover-controlled trial suggest that metabolic conditions in response to different carbohydrate supplements (Glu, Fru, GluFru, or sucralose) in healthy individuals are not associated with significant differences in HRV during CPET, as well as their CPET performance data analyses, including HRTP and time to HRTP. Heart rate response during exercise, HRV values, PVC, and QT interval analyses are known relevant clinical topics, whereas small alterations can cause severe, life-threatening cardiac supraventricular and ventricular arrhythmias as well as cardiovascular events [2,4].

Previous research on the impact of elevated blood glucose levels after glucose load revealed an increased sympathetic and decreased parasympathetic tone in subjects with metabolic disorders, suggesting an imbalance of the autonomic cardiac modulation in these subjects resulting from reactive hyperinsulinemia due to hyperglycemia [4]. In contrast to these findings, the results of this study highlight the preserved autonomic cardiac modulation in healthy young individuals being exposed to variable metabolic conditions. No adverse cardiac events from glucose and fructose supplementation were demonstrated in our cohort. Previous research on the consumption of energy drinks with similar ingredients has reported negative adverse effects, especially supraventricular extrasystoles (SVES) which were not shown during our study [10]. In contrast to our short time exercise guided observational analyses, Cirincione et el. investigated the influence of glycemic changes on transient QT interval change within a 24 h assessment by ECG monitoring, plasma glucose, and insulin concentration assessment [21]. These alterations might be triggered by the temporal delay of the incorporated meals as well as received medication influencing postprandial glucose concentrations [21]. These observations might contribute to electrical instability in these individuals, resulting in polymorph ventricular complexes (PVCs), SVES, and baseline ECG alterations, such as QT interval prolongation and subsequent clinical arrhythmogenic impact and adverse cardiac events. For these underlying conditions, reduced HRV in cardiac-affected patients has been demonstrated as a negative predictor of increased risk of cardiovascular mortality and cardiovascular events which was also not shown in our cohort [12]. Additionally, an inverse association between HRV and mass of body fat as well as a significantly higher HRV in physically active subjects have previously been reported, supporting our findings of preserved autonomic cardiac modulation in healthy young subjects independent of transiently acquired metabolic conditions [2].

Data from this study are in line with the previous findings of Birnbaumer et al. regarding the HRPC [22]. The S-shaped HR curve (Figure 3) found during CPET described previously [23] indicates a regular HR response to incremental resistance during CPX testing on a cycle ergometer. When investigating the heart rate curve during constant exercise tests following the ingestion of Glu, Fru, GluFru, or sucralose, the time until reaching the HRTP was similar. Even though participants received supplements inducing a metabolic response, the performance curve in those individuals remained similar [15]. From this aspect, this is the first study to show in detail that consuming carbohydrates directly prior to exercise until exhaustion has no detrimental effect on HR-performance in healthy subjects. Conconi et al. postulated that HR deflection could be induced metabolically which was questioned by Hofmann et al. previously and can also not be confirmed by our study results [24,25].

Our study is not without limitations. Firstly, the number of participating subjects was small, so the observed results have to be considered as hypothesis generating and have to be confirmed with more participants. This also did not allow a subgroup analysis between female and male participants of the study which could have delivered valuable information about the gender-specific responses to Glu, Fru, GluFru, and sucralose and performance. Future studies should aim for these measurements to close the gap in research regarding this important topic. Secondly, in our participating cohort, variable influencing factors, such as interindividual sleep hours or sleep deprivation, lifestyle habits, and variable physical activity which has not been standardized in our population have to be considered for their impact on HRV [2]. Although laboratory-based measurements can effectively assess the individual subject’s performance, general conclusions drawn from these results might not be fully applicable to routine daily conditions. In addition, our findings might not be applicable to individuals with metabolic disease, since their utilization of carbohydrates is altered.

Our study revealed that supplementation of Glu, Fru, GluFru, and sucralose prior to strenuous exercise seems to be safely applicable in healthy subjects. No association with adverse changes in HRV and baseline ECG alterations such as QT interval prolongation and negative impact on the HRTP and HR curve analyses were demonstrated. These findings might be useful for proving the feasibility of different combined carbohydrate supplementations in healthy young physically active individuals during sports activities and provide information for HRV, cardiac arrhythmogenic, and cardiac stress assessment in general. 

## Figures and Tables

**Table 1:** Resting 12-lead ECG.

Participant	HR (bpm)	PQ (ms)	QRS (ms)	QT (ms)	QTc (ms)	Sokolow (mm)	Lewis (mm)	Cornell (mm)	Electrical Axis
1	78	136	84	406	464	9.4	−5.6	6	Vertical
2	92	126	80	362	448	17.1	−6	3.2	Vertical
3	60	154	90	422	422	27.3	−4.8	4	Vertical
4	67	124	72	412	434	22.6	−12	0.4	Vertical
5	48	176	104	458	409	17,4	−6	8.6	Vertical
6	100	174	84	326	420	8.8	2	9.3	Left
7	72	158	106	400	439	25	−1.8	9	Vertical
8	77	154	70	374	424	22	10.5	6.1	Indifferent
9	73	120	74	382	422	24	2	5	Indifferent
10	53	124	106	450	424	24.5	−6.6	12.4	Vertical
11	57	162	68	444	434	26.4	−10.2	1.4	Vertical
12	60	144	110	426	427	45.2	−19.6	1.4	Vertical

HR: heart rate.

## Data Availability

The data is available upon reasonable request.
